# Assessment the relationship between health anxiety and iranian nurses’ quality of life: a cross-sectional study

**DOI:** 10.3389/fpsyt.2025.1447816

**Published:** 2025-03-27

**Authors:** Zahra Amoozadeh, Yazdan Ahmadi, Amir Hosein Pishgooie, Mehdi Fallah Bagher Shaidaei, Fatemeh Rumyani, Reza Momen, Masoud Rezaei

**Affiliations:** ^1^ Faculty of Nursing, Aja University of Medical Sciences, Tehran, Iran; ^2^ Emergency Nursing Department, Nursing School, Aja University of Medical Sciences, Tehran, Iran; ^3^ Ph.D. in Nursing, Professor, Critical Care Department, Faculty of Nursing, Aja University of Medical Sciences, Tehran, Iran; ^4^ Clinical Research Development Unit, Ganjavian Hospital, Dezful University of Medical Sciences, Dezful, Iran; ^5^ Student Research Committee, Nursing School, Aja University of Medical Sciences, Tehran, Iran; ^6^ Critical Care Nursing Department, Faculty of nursing, Aja University of Medical Sciences, Tehran, Iran; ^7^ Nursing and Midwifery Care Research Center, School of Nursing and Midwifery, Iran University of Medical Sciences, Tehran, Iran; ^8^ Cardiovascular Nursing Research Center, Rajaie Cardiovascular Medical and Research Center, Tehran, Iran

**Keywords:** health anxiety, quality of life, nurses, military nurse, occupational stress

## Abstract

**Background:**

Nurses are one of the most important pillars of any health organization, and their mental health is of great importance. Health anxiety is one of the factors that affect the psychological health of nurses and the presence of this disorder among nurses will change their quality of life.

**Objective:**

This study was conducted with the aim of investigating the level of health anxiety and its relationship with the quality of life of nurses working in selected hospitals of Aja University of Medical Sciences, Tehran, Iran.

**Methodology:**

In this descriptive-analytical study, 257 nurses working in different departments of selected Aja hospitals were randomly selected and their health anxiety levels were measured using the short version of Salkoskis and Varik’s Health Anxiety Questionnaire and their quality of life was measured using World Health Organization quality of life questionnaire was evaluated. Finally, the relationship between health anxiety and nurses’ quality of life was evaluated.

**Results:**

257 nurses who met the study entry criteria were randomly selected. Their mean health anxiety was evaluated as 35.21; This statistic shows that nurses’ health anxiety is at an average level. In addition, health anxiety was negatively associated with total score of the QOL (r = - 0.18, P = 0.003). Moreover, this relationship was significant for physical health (r = -0.14, P = 0.01), psychological health (r = -0.30, P <0.001), social relationships (r = -0.31, P <0.001), and social environment(r = -0.21, P <0.001).

**Conclusion:**

This study showed that there is a significant relationship between health anxiety and different dimensions of quality of life, so that the quality of life of nurses decreases with the increase of health anxiety.

## Introduction

Job-related stress is a significant issue, especially in healthcare professions like nursing (1). Nurses face various stressors such as continuous exposure to sick patients, dealing with death, heavy responsibilities, rapid advancements in technology, and infection risks (2, 3). These factors, along with interactions with patients, contribute to mental pressure and anxiety among medical staff (4). Occupational stress not only impacts the quality of nursing work but also correlates with mental health issues and potential physical illnesses (5). So that the results of a study in Iran showed that 38% of Iranian nurses reported poor oral health (6). Health anxiety, a common disorder in healthcare professions, involves excessive fear of serious illness (7). Individuals with health anxiety tend to hyper focus on bodily sensations, often misinterpreting them as signs of a severe ailment (8). This disorder can impair daily functioning and relationships (7, 9). People with health anxiety often adopt coping strategies like frequent doctor visits and excessive online research. The stress of constant patient interaction can significantly affect nurses’ quality of life (10). To ensure optimal patient care, nurses must maintain good mental health, job satisfaction, and overall well-being (11). Military nurses, facing additional stressors from military environments, may experience unique challenges compared to their counterparts in public hospitals (12). This study aims to explore the levels of health anxiety and domains of quality of among military nurses affiliated with Aja University of Medical Sciences in Tehran, Iran, shedding light on the interplay between these factors.

## Materials and methods

### Study population

This study is a descriptive analytical research conducted after following registration in the Behsan system and approval from the research council and ethics committee of Aja University. Upon obtaining written consent, data on the health anxiety and quality of life of nurses were collected using standard questionnaires. This correlational study involved nurses aged between 22 and 55.

#### Sampling method

Data collection from all Aja hospitals in Tehran employed a simple random sampling method to ensure a more accurate representation of the sample population. Initially, a comprehensive list of all Aja hospitals was compiled, and the data of their nurses were recorded. Each hospital was then assigned a random quota based on the number of nurses, ensuring a representative sample from each institution. Between September and December 2022, a total of 268 initial samples were collected; however, information from 11 participants was discarded due to incomplete questionnaires. Ultimately, 257 eligible nurses were selected for research. Participants were sourced from Aja University of Medical Sciences-affiliated hospitals, including Imam Reza, Golestan, Hajar, Bethat, Family, and No. 502, which serve as educational centers treating various patients, including military personnel from Iran’s provinces. These hospitals offer a wide range of healthcare services, including general, intensive care, and oncology.

#### Inclusion criteria

Inclusion criteria stipulated participants must hold at least a bachelor’s degree, be personally satisfied and willing to join the study, have worked in a clinical department for a minimum of 6 months, work in selected hospitals’ clinical departments, refrain from sleep, anti-anxiety, and sedative pills, have no history of anxiety disorders, and not have experienced major crises like the death of close family members or divorce.

#### Exclusion criteria

Exclusion criteria involved unwillingness to continue the study or incomplete questionnaire submissions.

#### Data collection

After obtaining informed consent from participants to take part in the study and explaining the study’s objectives, the Health Anxiety Questionnaire was provided to them. Following the completion of this questionnaire, the Quality of Life Questionnaire was also provided to the participants. The researcher addressed any questions that participants had and ensured that participants did not submit incomplete questionnaires. Nonetheless, a few samples of the questionnaires were submitted incomplete, and these incomplete data points were excluded from the study. Overall, the collected data included demographic information such as age, gender, education, economic status, marital status, and smoking habits, gathered through direct interviews with participants. Additionally, work-related details, including work experience, type of shifts (morning, evening, night, or rotating shifts), and average weekly working hours were also recorded. This data collection method allowed researchers to systematically gather quantitative data and analyze the potential relationships between health anxiety and the quality of life of nursing staff.

### Assessment of health anxiety

The Health Anxiety Inventory (HAI), a tool utilized in this study to assess nurses’ health anxiety levels, was developed by Salkosis and Warwick in 2002. It comprises 18 questions rated on a 4-point Likert scale from 0 to 3, yielding a maximum score of 54 and a minimum of 0. The HAI demonstrated high retest reliability (0.90) and acceptable internal consistency (Cronbach’s alpha ranging from 0.70 to 0.82), as reported by Salkoskis and Varik. To evaluate validity, researchers employed Illness Attitudes Scale (IAS), establishing the HAI validity at 0.63 (9, 13). In this study, the Persian version’s abbreviated form, validated in Iran by Nargesi et al., was adopted. Nargesi and colleagues confirmed the psychometric robustness of the Persian short health anxiety questionnaire, with a Cronbach’s alpha coefficient of 0.75 (13).

#### Quality of life assessment

The Persian version of the World Health Organization’s quality of life questionnaire^1^ was utilized to assess nurses’ quality of life. This condensed questionnaire evaluates physical health, mental health, social relations, and environmental health through 26 questions. Respondents indicate their level of agreement or disagreement on a five-point Likert scale from very good (5 points) to very bad (1 point). Salehi Omran’s research validated this questionnaire’s reliability and validity among the Iranian population, with a confirmed Cronbach’s alpha of 0.72 and validity across all domains (14).

### Statistical analysis

The statistical data obtained from the health anxiety questionnaire and the quality of life Inventory in order to determine the statistical relationship between the characteristics of health anxiety and quality of life of nurses using SPSS-24 software and statistical models of Fisher, Mann-Whitney and Chi-square was checked with an error rate of less than 0.05.

### Ethical considerations

This study received approval from the ethics committee (ethics code: IR.AJAUMS.REC.1401.154) at Aja University of Medical Sciences. The research objectives and procedures were elucidated to participants, emphasizing the confidentiality of all data. Subsequently, written informed consent was obtained from all individuals. Participants were informed that their involvement was voluntary, and they were guaranteed the privacy of their data with individual anonymity under a single identification code.

## Results

The demographic characteristics of the participants across different health anxiety score categories are outlined in **Supplementary Table S1**. The majority were male nurses (n=62.6), single (53.7%), with an average age of 31.66 years, and working rotating shifts (51%). Their mean health anxiety score stood at 16.82. The breakdown of participants in health anxiety categories was as follows: 48.6% in the low anxiety category, 30.4% in moderate anxiety, 13.2% in severe anxiety, and 7.8% in very severe anxiety. In the very severe health anxiety category (**Table 2**), the mean (SD) quality of life scores was MEAN (SD), with scores for physical health, psychological health, social relationships, and environment at 59/94 (11/58), 67/83 (15/97), 65/80 (14/68), and 63/06 (16/77) respectively. Details on the average quality of life scale and its dimensions can be found in **Table 2**.

According to **Table 1**, the results of Chi-square test showed that there was a significant difference between health anxiety and the marital status of nurses (P=0.03), and the results of Fisher’s exact test showed that there was a difference between the level of health anxiety and the level of education of nurses. So that nurses who had a Basic science reported a higher level of health anxiety than nurses who had a master’s degree (P=0.03). This difference was observed in the level of health anxiety and the economic status of the research units, so that nurses who had medium and medium to high economic status reported a lower level of health anxiety (P=0.04).

**Table 1:** Relationship between health anxiety and participant demographic characteristics.

Demographic Variables	Variable Levels	Total (257)	Test Statistics	p-value
Gender	Male	161	X^2^ = 0/96	0/82
	Female	96		
Marital status	Married	138	X^2^ = 8/58	0/03***
	Single	119		
Education	Basic science	227	X^2*^ = 11/61	0/03**
	Master Degree	28		
	PhD	2		
Ward	General	87	X^2^ = 3/91	0/69
	Emergency	55		
	Intensive care unit	115		
Smoking	Yes	210	X^2^ = 2/20	0/54
	No	47		
Working Shift	Morning	52	X^2^ = 3/93	0/91
	Evening	23		
	Night	51		
	Rotation	131		
working Average (hour/week)	Less than 35	28	X^2^ = 9/92	0/35
	36 to 45	115		
	46 to 55	38		
	56 or higher	76		
Economic Status	Weak	34	X^2^ =21/07	0/04****
	Medium to low	57		
	Medium	137		
	Higher than average Excellent	27		
		2		

*Fisher's Exact test.

**There is a significant relationship between education level and health anxiety.

***There is a significant relationship between marital status and health anxiety.

****There is a significant relationship between economic status and health anxiety.

The results of **Table 2** show that there is a significant relationship between health anxiety and quality of life and each of its different dimensions. So that health anxiety was negatively associated with total score of the QOL (*r* = - 0.18, *P* = 0.003). Moreover, this relationship was significant for physical health (*r* = -0.14, *P* = 0.01), psychological health (*r* = -0.30, *P <*0.001), social relationships (*r* = -0.31, *P <*0.001), and social environment (*r* = -0.21, *P <*0.001).

**Table 2:** The association between different domains of quality of life and Health Anxiety.

Quality of life (WHOQOL) and Related Domains	Mean	SD	Health Anxiety Mean: 16.82 SD: 9.10	
			r	P
Total QOL	69.33	27.35	-0.18	0.003
Physical Health	59.94	11.58	-0.14	0.017
Psychological Health	67.83	15.97	-0.3	<0.001
Social Relationships	65.8	14.68	-0.31	<0.001
Social Environment	63.06	16.77	-0.21	<0.001

Health Anxiety was based Health Anxiety Inventory of Salkovskis and Warwick; WHOQOL, The World Health Organization Quality of Life assessment; SD, Standard deviation.

## Discussion

The primary objective of this study was to investigate health anxiety and its correlation with the quality of life among nurses at selected Aja hospitals in Tehran, Iran. Findings revealed that the average age of nurses at Aja hospitals was 31.61 years, indicating a predominantly youthful workforce. The average tenure of these nurses was 10.09 years, reflecting a relatively shorter period due to some being part of general duty service in military hospitals (working in army hospitals as a conscript). Notably, 88.3% of nurses had attained education only up to the bachelor’s level. Recognizing this, aligning job roles with educational qualifications and promoting managerial positions for nurses with advanced degrees may incentivize pursuing master’s and doctoral studies.

Nurses play a crucial role in the healthcare system. They form the largest group of healthcare providers in hospitals, serving as the backbone of patient care. Their primary goal is to uphold and enhance the quality of care provided (15). The nursing profession encompasses a blend of technical expertise, professional competence, interpersonal skills, and empathy, each carrying significant weight and responsibility (16, 17). Alongside their core duties, nurses often contend with health-related stress stemming from their work environment (8). This health anxiety can influence their perception of stress, impacting their overall well-being and job performance (18). Health is a cornerstone of peace and well-being, directly influencing one’s ability to care for themselves and their loved ones. Health-related concerns frequently trigger anxiety, a natural response to potential harm (19). Health anxiety poses a substantial challenge, impacting individuals’ quality of life and exerting significant costs on society’s healthcare and well-being.

The primary objective of this study was to ascertain the mean score of health anxiety among nurses. The findings revealed that the mean score and standard deviation for health anxiety were 16.9 ± 10.82, with 48.6% of nurses exhibiting low levels of health anxiety. Moreover, only 21% of nurses were found to have severe or very severe levels of health anxiety. These results were contrary to the results of several previous studies. Research by Lai, Abdi, Babaei, and Santoz suggested that nurses typically face high levels of health anxiety (8, 20–22). Mousavi et al.’s study, conducted during the coronavirus pandemic, indicated that the majority of nurses (91%) experienced moderate health anxiety levels (23). Isik’s study, involving 826 nurses in Turkish hospitals during the pandemic, revealed that 78% of nurses reported health-related anxiety, with 89% attributing this stress and anxiety to fear of infecting their family members (24). Health anxiety appears to encompass critical health-related concerns affecting individuals of all ages and is shaped by diverse socio-economic, psychological, and managerial factors (25). Discrepancies between this study and others may be attributed to Babaei and colleagues’ comparison of nurses’ health anxiety levels with those of the general population, as well as variations in the timing of research conduct. Previous studies were undertaken prior to or during the coronavirus outbreak, comparing nurses’ health anxiety levels with those of the general population. In contrast, the present study was conducted post-coronavirus pandemic, amid the introduction and administration of effective vaccines against COVID-19, resulting in a relatively secure environment and peace of mind after the critical circumstances of the pandemic. It is plausible that these factors have fostered a positive outlook, diminishing worries, fears, and health anxiety among nurses. On the other hand, it can be said that individuals visiting military hospitals belong to a specific segment of society and differ from patients visiting other public hospitals in Tehran in terms of education level and lifestyle. Additionally, the number of individuals visiting these centers is lower compared to other facilities. Given that workload can impact the health anxiety of nurses, it can be concluded that one reason for the differences in the results of the current study compared to other studies may be related to the work environment and the demographic characteristics of patients visiting military treatment centers.

The second objective of this study was to ascertain nurses’ mean score of quality of life. Quality of life represents an individual’s perception of their personal life circumstances, culture, and values within their environment (16). Findings of this research revealed that the mean and standard deviation of nurses’ overall quality of life score was 69.33 ± 27.35, with 50.6% of nurses falling into the category of average quality of life. Furthermore, the mean and standard deviation for the physical health dimension score were 59.94 ± 11.58, for psychological health it was 67.83 ± 15.97, for social relations it was 65. 80 ± 14.68, and for social environment it was 63.06 ± 16.77. Notably, the psychological health dimension scored the highest average, while the physical health dimension scored the lowest. Overall, nurses reported an average quality of life across these dimensions. These outcomes align with Orszulak’s investigation. Orszulak et al.’s study on 312 nurses in Poland concluded that nurses rated their quality of life as average, recording the highest score in psychological health and the lowest in physical health (26). Nurses’ quality of life is subject to change due to challenges and work-related stress (27). The average quality of life among nurses in military hospitals can be attributed to factors such as unique organizational circumstances, experience in challenging conditions and crises, modest expectations, and high job security within military settings. In another research reveals that better remuneration and benefits in civilian sectors tempt nurses to transition from military roles (28). Additionally, army nurses’ average quality of life may stem from their focus on psychological and social dimensions as indicated by the World Health Organization quality of life questionnaire. Reflecting similarities with this study, Mohammadzadeh et al.’s investigation comparing nurses’ quality of life in coronavirus referral centers with non-coronavirus facilities in Sabzevar city showed that 58.3% of nurses had average quality of life (29). Furthermore, Veismradi et al.’s study on 360 nurses in Kermanshah hospitals revealed that the majority had an average quality of life, scoring an average of 59.70 (30). Similarly, Inousian et al.’s study during the same period as the Corona pandemic in Saudi Arabia found nurses to have an average quality of life (31), consistent with our findings. Baronizadeh et al.’s 2011-2012 assessment categorized nurses’ quality of life as good (32). Orszulak et al. research on 312 Polish nurses reported that over 56% rated their quality of life as good (26). Disparities in quality of life findings across studies could be attributed to distinct social and cultural contexts and varying working conditions for nurses. Jodaki et al.’s research in Khorram Abad city noted that 60.5% of nurses experienced poor quality of life. Nonetheless, when assessing quality of life across dimensions, the results echoed those of the present study. Jodaki attributed the reduced quality of life in his study to unique environmental factors and escalated tensions between nurses and patients, particularly among female and married nurses (33).In general, it can be said that the quality of life of nurses may vary in different caregiving environments or even among various ethnic cultures in Iran. For example, in smaller cities with lower populations, nurses do not have to spend more hours in urban traffic to reach their workplaces, whereas in Tehran, conditions such as traffic congestion and high living costs can significantly affect the quality of life of caregiving personnel.

The study’s third objective was to establish the association between health anxiety and nurses’ quality of life. Findings indicated a significant inverse relationship between health anxiety levels and nurses’ quality of life (P=0.003, r=0.18), suggesting that heightened health anxiety diminishes their quality of life. Furthermore, a significant negative association was observed between health anxiety and all facets of quality of life, indicating that elevated health anxiety among nurses corresponds to reduced quality of life across all dimensions. Conversely, it can be argued that nurses with higher quality of life experience lower levels of health anxiety. These outcomes are consistent with Ozturk, Celmece, and Mazhari’s research, highlighting the detrimental physical and mental consequences of heightened anxiety on health and quality of life (34–36). A study involving 250 female nurses in Ahvaz revealed that anxiety stemming from the coronavirus outbreak adversely affected nurses’ quality of life. Stress and anxiety are natural responses to challenging situations like the COVID-19 pandemic; while beneficial in the short term, prolonged presence can impact an individual’s quality of life (34). These results are in line with Mohammadzadeh et al.’s research findings (29). One of the possible reasons for this inverse relationship is the impact of anxiety on the psychological health and job performance of nurses. Health anxiety can lead to feelings of insecurity, decreased concentration, and increased fatigue, which in turn negatively affect the job performance and social relationships of nurses. On the other hand, nurses who have a higher quality of life generally benefit from better social support, more favorable working conditions, and stronger mental health, which can help reduce their levels of anxiety.

The fourth aim of this research was to determine the relationship between health anxiety and demographic characteristics of nurses the results showed that there was a significant difference between health anxiety and marital status of nurses (P=0.03) and married people had a higher level of health anxiety. More health anxiety among married nurses can originate from the responsibilities of married life. The results of Celmece study revealed that married nurses have higher health anxiety. Married nurses, in addition to caring in the hospital, are also responsible for doing things at home and raising and taking care of children, and high work pressure causes stress and anxiety and may even cause a person to quit their job (36). The results of Santoz’s study in Brazil during the time of Corona showed that married nurses have a higher level of anxiety and depression than single people. He believes that the fear of family members getting infected with covid-19 is the reason for higher mental disorders such as stress and anxiety in married people (22). In Isik’s research, it was discovered that worrying about transmitting the covid-19 infection to family members, fear of losing family members, and the inability to fulfill the family’s social needs were the primary reasons for anxiety among nurses during the covid-19 pandemic (24). Also, the results showed that there was a difference between the level of health anxiety and the level of education of nurses (P=0.03) and nurses who had a bachelor’s degree reported a higher level of health anxiety than nurses who had a master’s degree. There was a significant difference between the level of health anxiety and the economic status of the research units, and nurses who had a medium and medium to high economic status had a lower level of health anxiety. Similar results in the study by Que et al. in China showed that having more family income and doing physical activities in nurses reduced their anxiety and depression during the Corona period (37). But in the study of Santos in Brazil on 490 nurses, it was found that although nurses who had more income had less anxiety, there was no significant relationship between the amount of monthly income and the anxiety of nurses during the corona pandemic (P=0.38) (22).

The final goal of this study was to determine the relationship between quality of life and the demographic characteristics of nurses. The results of the Chi-square test indicated a significant difference between the psychological health aspect and the gender of nurses (P=0.02), with a high level of psychological health for men at 32.3% and for women at 24.2%. In the study by Orsolak, no significant relationship was found between quality of life and the gender of nurses (P>0.05), which may be attributed to the substantial difference in the number of female and male nurses participating in this research (297 women and only 15 men) (26). Female nurses are more affected by unfavorable working conditions due to heavy responsibilities as mothers, raising children, housekeeping, and greater sensitivity to their physical and mental well-being. The weakening of family relationships, distancing from social networks, and social isolation during the COVID-19 pandemic likely had a more negative impact on women, leading to changes in their psychological health and quality of life (30). Additionally, the results of the Chi-square test showed a significant difference between quality of life and educational level in the study units (P=0.017), with individuals holding a master’s degree reporting a higher quality of life than nurses with a bachelor’s degree. There was also a significant difference between the psychological health aspect and the educational level in the study units (P=0.006), with nurses holding a master’s degree demonstrating higher psychological health. The Chi-square test results indicated a significant difference between quality of life and work shifts in the study units (P=0.01). 51.9% of morning shift nurses reported a high quality of life, whereas rotating shift nurses reported the lowest quality of life at 24.4%. This indicates that stress associated with rotating shifts reduces individual quality of life. According to Santos’s study in Brazil, 71% of nurses preferred to work during the day, and many of them had less willingness to work rotating and night shifts due to the negative impact of night shifts on quality of life (22). In this study, there was a significant difference between the social environment aspect and work shifts in the study units (P=0.005). The social environment was reported to be highest among morning and evening shift nurses, with a frequency of 32.7%. The Chi-square test results indicated a significant difference between quality of life and the average weekly work hours of nurses (P=0.001). Nurses working less than 35 hours per week had the highest quality of life, whereas those working more than 55 hours had the lowest quality of life. This study also determined a significant difference between quality of life and the average hours of sleep per day (P=0.01), indicating that nurses who sleep less than 6 hours or more than 10 hours per day experienced lower quality of life. Furthermore, the results showed a significant difference between quality of life and the economic status of nurses (P<0.001), with those in better economic situations reporting a higher quality of life, consistent with the studies by Santos and Orsolak (22, 26). In the study by Santos and colleagues in Brazil, aimed at exploring the relationship between quality of life and workplace factors among nurses, it was found that 69.9% of nurses were not satisfied with their salaries, and there was a significant relationship between income and their quality of life (p=0.001) (38). If nurses do not enjoy a desirable quality of life and job satisfaction, the first adverse effects will reflect on the patient status. Therefore, considering that nurses are the largest service-providing group in the healthcare system, they must maintain a satisfactory quality of life to provide optimal care to patients. This is only possible if nurses are at an optimal level physically, mentally, socially, and in terms of job satisfaction and various aspects of life (11).

## Conclusion

In conclusion, this study clearly demonstrates a significant inverse relationship between health anxiety and the quality of life of Iranian nurses. The results indicate that increased health anxiety has considerable negative effects on the mental well-being and job performance of nurses, highlighting the need for systemic changes in healthcare environments. Furthermore, future research could focus on longitudinal studies, examining the impact of specific interventions to reduce health anxiety, as well as analyzing influential factors such as social support, to enhance the quality of life for nurses and improve patient care.

### Limitations of study

Limitations are an inseparable part of any research. Despite efforts to minimize them, certain factors may challenge the interpretation of the study’s findings. The cross-sectional design of the study and its restriction to nurses working in military hospitals in Tehran may limit the generalizability of the results. Additionally, the use of self-reported questionnaires could have introduced inaccuracies due to participant fatigue, potentially affecting the precision of responses. The study also lacked control over the nurses’ mental and emotional states, which may have been influenced by personal life events. Furthermore, various external factors, such as managerial style, workplace relationships, job satisfaction, and occupational stress, could have impacted the study’s findings.

### Practical implications for nurses and healthcare

The findings highlight the need for healthcare systems to implement enhanced support mechanisms for nurses, particularly in addressing health anxiety. This could include access to mental health resources, counseling services, and peer support groups. Also Developing and incorporating training programs focused on managing health anxiety and promoting self-care strategies could be beneficial. This would empower nurses to maintain their mental well-being and improve their overall quality of life.

## Data Availability

The original contributions presented in the study are included in the article/**Supplementary Material**. Further inquiries can be directed to the corresponding author/s.

## Footnotes

1 - WHO-QOL-BREF
