# Supplementary material for: Assessment the relationship between health anxiety and iranian nurses’ quality of life: a cross-sectional study
Source: Front Psychiatry. 2025 Mar 27;16:1447816. doi: 10.3389/fpsyt.2025.1447816 (PMC11983459; doi:10.3389/fpsyt.2025.1447816)
Supplement: Supplementary file 1 [file Table1.docx]

| # | Title | Authors  (Year) | Journal | Methodology/ study design/ setting | Papulations/ Sample Size | AI Technology | Outcomes | Nursing Implications | Limitations |
| --- | --- | --- | --- | --- | --- | --- | --- | --- | --- |
| 1 | Decision support system for triage management: A hybrid approach using rule-based reasoning and fuzzy logic | Dehghani, et al  (2018) | Int J Med Inform | hybrid approach (review)/The study used a hybrid approach combining rule-based reasoning and fuzzy logic to accurately determine the triage level of patients. Surveys and interviews were conducted with clinicians and technical experts to examine the efficiency of paper-based triage and to determine the major weak and strong points of the existing system. | clinicians (emergency medicine experts and triage nurses) | The study developed a CDSS (Clinical Decision Support System) for predicting triage output. | well-recognized solution to increasing  the quality and efficiency of care | The study has implications for nursing in emergency departments as it can improve the efficiency and quality of patient care. The CDSS can help nurses accurately determine the triage level of patients, reducing the risk of human errors and improving patient outcomes. | The study was conducted in a single hospital and may not be generalizable to other settings. The CDSS was not tested in a real-world clinical environment, and further research is needed to evaluate its effectiveness. |
| 2 | Nurses Seeing Forest for the Trees" in the Age of Machine Learning: Using Nursing Knowledge to Improve Relevance and Performance" | Kwon, et al  (2019) | Comput Inform Nurs | Case study /Application of different machine learning algorithms to the same hospital data set to predict early hospital readmission in patients with diabetes | 130 specialized hospitals with 101 766 patients with diabetes | Supervised machine learning algorithms - The scientific article discusses the application of supervised machine learning algorithms to predict early hospital readmission in patients with diabetes. | nurses can provide more visibility to nursing work, advance nursing science, and better individualize patient care. | Nurses should participate in all phases of development, implementation, and evaluation of machine learning algorithms to ensure they are shaped by nursing knowledge and standardized nursing terminologies are integrated. Developing better decision support tools that simulate expert nurses' processes is an active area of research. The use of machine learning in healthcare raises questions regarding the role of nurses and other clinicians in making clinical decisions based on machine predictions. | - |
| 3 | Artificial intelligence in healthcare in developing nations: The beginning of a transformative journey | Mahajan, et al  (2019) | Cancer Research, Statistics, and Treatment | review /The article provides a comprehensive review of the current status of Artificial Intelligence (AI) in healthcare, with a focus on radiology in developing nations like India. The authors discuss the potential of AI in early detection and diagnosis, and the need for ethical considerations and collaborations for its apt implementation. | Not Mention | The article discusses various AI technologies that can be used in healthcare, including machine learning, natural language processing, and computer vision. | ensure smooth transition and implementation of AI in healthcare | the article does mention the potential of AI to aid in the decentralization of medical services and bridge the gap for the need of specialized medical personnel in peripheral areas in developing countries like India. This could have implications for nursing in terms of providing access to healthcare services and improving patient outcomes. Additionally, the article emphasizes the need for ethical considerations and collaborations for the implementation of AI in healthcare, which could involve nursing professionals in decision-making processes. | - |
| 4 | Automatic learning for improvement in joint mobility in the elderly | Romero, et al  (2019) | Communications in Computer and Information Science | A narrative review /This article presents a narrative review of the available evidence on programs aimed at avoiding pain in the elderly. The review covers a period between the years 1999 to 2017 and includes databases such as PubMed, Dialnet, Cuiden, IME, LILACS, Cochrane Library Plus, ENFISPO, Medline, and Elsevier. The article discusses the methodology used in the review, including the descriptors used to search the databases and the review of book deposits located at the University of Castilla la Mancha. | Not Mention | This article discusses the use of a virtual agent based on computer vision, artificial intelligence (Machine Learning and Deep Learning), and natural interfaces to improve joint mobility in the elderly. The article highlights the benefits of using this technology, including the ability to personalize treatment plans and improve patient outcomes. The article also discusses the limitations of this technology, including the need for further research to determine its effectiveness. | joint mobility improvement | This article discusses the nursing implications of using automatic learning technology to improve joint mobility in the elderly. The article highlights the need for greater involvement of nursing specialists in geriatrics and gerontology, as well as the potential for reducing drug doses and unattended sessions for joint mobility improvement. The article also discusses the importance of patient education and the need for further research to determine the effectiveness of these interventions. | This article discusses the limitations of the study, including the exclusion of articles in languages other than Spanish and English and articles not found in full texts. The article highlights the potential for bias in the review due to these limitations and the need for further research to address these limitations. The article also discusses the importance of transparency in research and the need for researchers to report their limitations. |
| 5 | Ai chatbot design during an epidemic like the novel coronavirus | Battineni, et al  (2020) | Healthcare (Switzerland) | design of a sophisticated artificial intelligence / The article presents the design of a sophisticated artificial intelligence (AI) chatbot for the purpose of diagnostic evaluation and recommending immediate measures when patients are exposed to nCOV-19. The chatbot is designed to promote preventive measures, provide virus updates, and reduce psychological damage caused by isolation and fear. | Not Mention | The chatbot engine is being developed in Python, and Watson is used as the AIML platform | diagnostic evaluation and recommending immediate measures | it does mention that the AI chatbot can provide access to virtual doctors and health specialists, which can be beneficial for patients living in remote areas. Additionally, the chatbot can promote preventive measures and provide virus updates, reducing the psychological damage caused by isolation and fear. | - |
| 6 | Fall Detector Adapted to Nursing Home Needs through an Optical-Flow based CNN | Carlier, et al  (2020) | Annu Int Conf IEEE Eng Med Biol Soc | Stages:A. Solution overview, B. Databases and training procedure /The article proposes a vision-based fall detection solution for nursing homes using Deep Learning and a Convolutional Neural Network (CNN) trained to maximize a sensitivity-based metric. The solution is built on an Optical-Flow based CNN and is designed to detect falls without the need for wearable sensors. | Not Mention | Vision-based fall detection solutions using Deep Learning and a Convolutional Neural Network (CNN) trained to maximize a sensitivity-based metric | implementation of a decision-making process | Improved fall detection in nursing homes, without the need for wearable sensors, and with high accuracy and low false alarm rates | The study was conducted in three specialized homes for the elderly in the metropolitan area of Rennes, France, and may not be generalizable to other settings. The proposed solution requires the use of cameras, which may raise privacy concerns. |
| 7 | Role of Assistive Robots in the Care of Older People: Survey Study Among Medical and Nursing Students | Łukasik; et al  (2020) | J Med Internet Res | Survey Study /Quantitative Analysis of Survey Responses from Medical and Nursing Students | 178 students from Poznan University of Medical Sciences, Poznań, Poland  (110 nursing students and 68 medical students) | Assistive Robots for Elderly Care: Attitudes and Acceptance Among Future Medical Professionals | suited to their needs | The use of artificial intelligence (AI) in nursing practice and education has the potential to improve patient outcomes, increase efficiency, and enhance decision-making. However, there are also ethical concerns, data privacy and security issues, a lack of training and education, and cost considerations that must be carefully considered before implementing AI technology. | Small Sample Size and Lack of Practical Experience with Assistive Robots Limit the Generalizability of Study Findings |
| 8 | Improving breast cancer care coordination and symptom management by using AI driven predictive toolkits | Moser and . Narayan  (2020) | Breast | Original article /The article discusses the use of AI-driven predictive toolkits to improve breast cancer care coordination and symptom management. It highlights the importance of communication, digital tools, and multidisciplinary team meetings in breast cancer care. The article also emphasizes the role of nurse navigators in ensuring patient care continuity. | N/A | The article discusses the use of AI-driven predictive toolkits to improve breast cancer care coordination and symptom management. | clinical practice and drive well-coordinated, patient-centric cancer care | The article highlights the pivotal role of nurse navigators in ensuring patient care continuity and the importance of educating patients on early symptom detection. It also discusses the potential of automated filter tools and chatbots to assist patients in symptom reporting. | The article cautions that AI systems may get overwhelmed with data and emphasizes the need for continuous monitoring of the model implemented in clinical practice to avoid implications on patient care. It also notes that Artificial Neural Networks offer very little information about how they arrive at conclusions. |
| 9 | Novel e-health applications for the management of cardiometabolic risk factors in children and adolescents in greece | Tragomalou; et al  (2020) | Nutrients | Review /The authors reviewed various e-Health applications developed and implemented in Greece for the prevention and management of overweight and obesity in childhood and adolescence. They discussed the potential benefits and limitations of these applications and their implications for healthcare providers and policymakers. | Not Mention | The article focuses more on the potential benefits and limitations of these applications and their implications for healthcare providers and policymakers. However, the article does mention the development of intelligent multi-level information systems and specialized artificial intelligence algorithms for personalized management of obesity in childhood and adolescence as a project currently in progress in Greece. | computerized decision-support tool was developed to assist pediatric health care  professionals in delivering personalized nutrition and lifestyle optimization advice to overweight or  obese children and their families. | The article suggests that nurses can use these applications to monitor and manage the health of children and adolescents with cardiometabolic risk factors, and to provide personalized care and education to patients and their families. Nursing education programs can also incorporate these applications into their curricula to prepare future nurses for the use of technology in healthcare. | - |
| 10 | A natural language processing and deep learning approach to identify child abuse from pediatric electronic medical records | Annapragada;et al  (2021) | PLoS ONE | retrospective study /The study involved developing and evaluating NLP-based AI models that utilize free text from pediatric EMRs to classify suspected child victims into abuse-positive and abuse-negative groups, for potential work up by CAP team physicians. | Cases are referred to the CAP Team by clinical departments and  social workers within the hospital, county Child Protective Services, and community pediatricians  from the hospital’s satellite referral sites at five locations | The study utilized natural language processing and deep learning approaches to classify inflicted injury to children using only the portions of the electronic medical record before referral to a CAP team. | help screen patients/ identification of patients/ develop computer-aided-diagnosis | The study has important implications for nursing practice, as it highlights the need for nurses to be aware of the signs and symptoms of child abuse and to document them accurately in the electronic medical record. The use of AI technology can help nurses and other healthcare providers to identify cases of suspected child abuse more accurately and efficiently, which can ultimately improve patient outcomes. | The study acknowledges several limitations, including the need for further validation of the AI models in larger and more diverse patient populations, as well as the potential for bias in the data used to train the models. Additionally, the study notes that the AI models are not intended to replace clinical judgment or the need for a thorough evaluation by a multidisciplinary team. |
| 11 | How the nursing profession should adapt for a digital future | Booth; et al  (2021) | BMJ | literature review /The methodology used in this article is a literature review, where the authors examined various studies and articles related to the use of AI technologies in healthcare and synthesized the information to identify key themes and issues related to the use of AI in nursing practice. | 24 Articles | AI in general | digital technologies  already bring benefit to nursing practice and  education | Need for re-envisioning nurse-patient relationships, inquiry into AI's influence on human decision making and labor, need for policies on professional accountability, educational and leadership competencies, and opportunities related to AI and data analytics, ethical and accountability concerns, potential benefits in data analytics and advanced clinical decision support, and need for collaboration between technologists, researchers, providers, and users. | Lack of evidence for the purported potential benefits of AI in nursing research, potential for biases in current datasets to become ingrained in AI algorithms, unintentional reduction of nursing involvement in the development of AI systems, and potential for self-evolving algorithms to reinforce systemic inequities found in society. |
| 12 | Automatic Health Machine for COVID-19 and Other Emergencies | Ganesh;et al  (2021) | International Conference on Communication Systems and Net works, COMSNETS 2021 | survey /The study was conducted through a survey of healthcare professionals in India, and virtual workshops with COVID-recovered patients and frontline nurses and doctors to understand healthcare/patients’ requirements considering pandemic and post-pandemic. | Not Mention | The AHM uses Artificial Intelligence to improve speed and accuracy in treating patients, and to help nurses and doctors spend more time treating patients rather than getting details. The GUI-based first aid services and emergency button can save many lives as medications are initiated timely. | The healthcare professionals feel the system can be adopted in an area where medical facility is not available immediately. | The AHM can be deployed in rural primary healthcare centers, various locations at rural areas where healthcare facilities are very far, quarantined areas, hospitals, COVID testing camps, highways, and accident-prone areas. The system can be adopted in an area where medical facility is not available immediately, and it can be used in public settings such as a community center, jail, school, retail store, mall, office building, or airport. | The study was conducted only from healthcare professionals’ perspectives in India, and the prototype solution has not yet been fully tested in real-world settings. |
| 13 | Artificial intelligence for hospital health care: Application cases and answers to challenges in european hospitals | Klumpp; et al  (2021) | Healthcare | Survey /The study used a survey to identify the current challenges and areas of interest of European hospitals. Hospital decision-makers identified specific areas of application and presented the focus and expected output of the utility of AI. | European hospitals | The article discusses various AI technologies used in European hospitals, including machine learning, natural language processing, and computer vision. | Not Mention | The article discusses the potential benefits of AI in nursing, including improved patient outcomes, increased efficiency, and reduced workload. However, it also highlights the need for nurses to be involved in the development and implementation of AI technologies to ensure they align with nursing values and ethics. | The study is limited to European hospitals and may not be generalizable to other regions. Additionally, the survey used in the study may not have captured all relevant information about the use of AI in hospitals. |
| 14 | Utilization of Nursing Defect Management Evaluation and Deep Learning in Nursing Process Reengineering Optimization | Liu and Liu  (2021) | Comput Math Methods Med | Model Network Framework Based /The study uses statistical analysis and data mining to analyze nursing data from a macro and micro perspective. The Apriori algorithm is improved through simulation, and the study makes use of expert consultation and questionnaire survey methods. | 25 nursing staff | The study uses statistical analysis and data mining to analyze nursing data from a macro and micro perspective. The Apriori algorithm is improved through simulation, and the study makes use of expert consultation and questionnaire survey methods. | provides a new idea for in-depth learning in dealing with nursing defect management and is of great significance for the follow-up improvement of nursing process reengineering. | The study provides data support for decision-making departments to improve long-term nursing through data analysis and rule generation. It also shows that personalized nursing based on deep learning emotion classification model can reduce the degree of depression. | The study does not provide a unified opinion on how many items are retained in the consistency level according to the prepared item table. Additionally, the study's results are limited to the specific sample size, research purpose, and resources used. |
| 15 | Skin tear classification using machine learning from digital RGB image | Nagata;et al  (2021) | J Tissue Viability | The study used digital RGB images of skin tears to train and test support vector machine and random forest models for skin tear classification. The models were evaluated based on their accuracy in classifying skin tears into the appropriate STAR category. | 31 images | The study used machine learning algorithms, specifically support vector machine and random forest, to classify skin tears based on the STAR Skin tear classification system. | to aid nurses in their management of skin tears | The study suggests that machine learning algorithms can provide accurate classification of skin tears, which can help nurses correctly assess skin tears and provide standardized care even if they are not specialized in wound care. The STAR estimation algorithm is potentially useful for supporting healthcare providers in performing standardized evaluation and management of skin tears. | The study was conducted under a relatively controlled environment, and a feasibility test on a smartphone application will be required to assess the accuracy of the algorithm under various lighting conditions. Additionally, the study was conducted on a small sample size, and further research is needed to validate the algorithm on a larger sample size. |
| 16 | Application of artificial intelligence in community-based primary health care: Systematic scoping review and critical appraisal | Rahimi; et al  (2021) | Journal of Medical Internet Research | Systematic Scoping Review /The authors conducted a systematic scoping review and critical appraisal of studies that have tested or implemented AI in community-based primary health care settings. They used a PICOS format to describe the studies and performed a descriptive synthesis to summarize the results. The authors also consulted with a multidisciplinary group of experts to collect feedback on their preliminary results. | Community-Based Primary  Health Care | Various AI technologies were used in community-based primary health care, including machine learning, natural language processing, and computer vision. These technologies were used for various purposes, such as predicting patient outcomes, identifying high-risk patients, and improving diagnostic accuracy. | efficiently guide  the development and implementation of AI interventions | Artificial intelligence can be applied in nursing in various ways, such as predicting patient outcomes, identifying high-risk patients, and improving diagnostic accuracy. Nurses can play a key role in the implementation and use of AI technologies in healthcare, but it is important for them to be aware of the limitations and challenges of AI, such as ethical concerns and the need for ongoing training and education. | The authors noted several limitations of the scoping review, such as the exclusion of non-English language studies and the potential for publication bias. The quality of the included studies varied, which may have affected the overall findings of the review. |
| 17 | Content validation and usability of a chatbot of guidelines for wound dressing | Roque; et al  (2021) | International Journal of Medical Informatics | Methodological research was carried out in three phases : validation of the script’s content, develop chatbot, System Usability Scale/The study involved the development of a chatbot called BOTCURATIVO, which was designed to provide wound care guidance to both nurses and patients. The chatbot was evaluated through a survey and focus group discussions with nurses and patients. | 17 users | The chatbot was developed using artificial intelligence (AI) technology, specifically natural language processing (NLP) and machine learning algorithms. | Not mention | The study found that the chatbot was generally well-received by both nurses and patients, and had the potential to improve wound care management by providing accessible and reliable guidance. However, some concerns were raised about the chatbot's ability to handle complex cases and the need for ongoing training and support for nurses. | The study was limited by its small sample size and the fact that it was conducted in a single hospital setting. Further research is needed to evaluate the effectiveness of the chatbot in improving wound care outcomes and to address concerns about its implementation and use. |
| 18 | Capacity building in screening and treatment of diabetic retinopathy in Asia-Pacific region | Silpa-Archa; et al  (2021) | Indian J Ophthalmol | A systematic literature /The authors conducted a comprehensive search of the literature on DR screening and treatment, with a focus on studies that evaluated the use of nonophthalmologists, including nurses, in these processes. They also reviewed studies on the use of artificial intelligence (AI) in DR screening and treatment. | 6 Studies /diabetic retinopathy in Asia‑Pacific region | The authors reviewed studies that used AI technology in DR screening and treatment, including studies on the use of AI for glaucoma management and retinopathy of prematurity diagnosis. | N/A | The authors discussed the implications of their findings for nursing, including the potential for nurses to be trained to perform intravitreal injections of antivascular endothelial growth factors for DR treatment. They also highlighted the role of nurse-led clinics in DR screening and treatment, and the importance of appropriate training and guidance for nonophthalmologists who are involved in these processes. | lack of standardization in training and certification for nonophthalmologists who perform DR screening and treatment, and the need for further research on the safety and efficacy of these processes. They also highlighted the need for more studies on the use of AI in DR screening and treatment, particularly in low-resource settings. |
| 19 | Improving time to palliative care review with predictive modeling in an inpatient adult population: study protocol for a stepped-wedge, pragmatic randomized controlled trial | Wilson; et al  (2021) | Trials | study protocol /The study aims to improve time to palliative care review with predictive modeling in an inpatient adult population. The trial will be conducted in four nursing units of two hospitals in the United States. The study will use a machine learning algorithm to identify patients who may benefit from palliative care. | Saint Mary’s Hospital & Methodist Hospital both within Mayo Clinic Rochester in Minnesota. | The study will use a gradient boosting machine (GBM) algorithm to predict the likelihood of a patient benefiting from palliative care. | clinical practice while retaining scientific rigor. | The study has implications for nursing practice, as it aims to improve the quality of life of patients with complex or life-threatening illnesses by identifying those who may benefit from palliative care. Nurses will play a key role in implementing the algorithm and ensuring that patients receive appropriate care. | The study acknowledges that there are limitations to the use of AI in healthcare, including the need for validation studies and the complexity of deploying an algorithm in a clinical setting. The study also notes that the development of a large interdisciplinary team can be difficult to create and maintain. |
| 20 | Use of a Social Robot (LOVOT) for Persons With Dementia: Exploratory Study | Dinesen; et al  (2022) | JMIR Rehabil Assist Technol | Exploratory Study /This article describes an exploratory study that aimed to investigate the interaction between the social robot LOVOT and persons with dementia, as well as the experiences of healthcare professionals working with LOVOT in their interaction with persons with dementia. The study was conducted at three nursing homes in Denmark, and a triangulation of data collection techniques was used, including questionnaires, face scales, participant observation, and semi-structured focus group interviews. The study included both individual and group sessions, and the outcome measures included well-being, impact on mood and behavior, acceptance of LOVOT, and LOVOT's interaction with persons with dementia. | 3 nursing homes in Denmark/ 42 persons | LOVOT, a social robot | facilitated interpersonal interaction | The study suggests that social robots may have the potential to improve the quality of life for persons with dementia and reduce caregiver burden, which could have positive implications for nursing practice. However, the study also notes that further research is needed to fully understand the impact and limitations of this technology in healthcare settings. Overall, the use of artificial intelligence in nursing is an emerging area of research and practice, with potential applications in areas such as patient monitoring, decision-making, and education. | Small sample size, limited generalizability, and potential bias from using healthcare professionals as observers.  These features suggest that the robot may use machine learning algorithms or other forms of AI to process and respond to sensory input. |
| 21 | Addressing Mild Cognitive Impairment and Boosting Wellness for the Elderly through Personalized Remote Monitoring | Ianculescu; et al  (2022) | Healthcare (Switzerland) | review /The study uses a personalized remote monitoring system that collects data from various sensors and devices to monitor the cognitive and physical health of elderly individuals. The collected data is then analyzed using machine learning algorithms to detect any changes in behavior or activity patterns that may indicate cognitive decline. | Not Mention | The study uses various machine learning algorithms, including decision trees, random forests, and support vector machines, to analyze the collected data and detect any changes in behavior or activity patterns. | continuously monitor patient | The study has important implications for nursing and healthcare professionals who work with elderly individuals. The personalized remote monitoring system can help nurses and healthcare professionals detect early signs of cognitive decline and intervene before the condition worsens. This can improve the quality of life for elderly individuals and reduce the burden on caregivers. | The study has some limitations, including the small sample size and the fact that the study was conducted in a controlled environment. The authors note that further research is needed to validate the effectiveness of the personalized remote monitoring system in real-world settings. |
| 22 | Digital technologies and the role of health care professionals: scoping review exploring nurses’ skills in the digital era and in the light of the COVID-19 pandemic | Isidori; et,al  (2022) | JMIR nursing | Scoping Review /The methodology of the article is a scoping review that used a thematic synthesis approach to analyze the literature published within a 10-year span (2011-2021) to identify emerging skills and attitudes required for nurses in the digitalized healthcare system, new responsibilities for healthcare staff, and the potential impact of digital innovation on nursing university curricula. | 26 Articles | AI in general | Increasing the quality and efficiency of care | it does discuss the impact of digital innovation on nursing and healthcare delivery. The authors highlight the importance of nurses mastering new methodological approaches and digital knowledge in a continuously evolving health care scenario that relies increasingly more on technology and digital solutions. They also discuss the need for nurses to develop new communication skills, adaptiveness, and problem-solving abilities to adapt the interaction to the level of digital skills and digital knowledge of the patient. While the article does not discuss the use of artificial intelligence in nursing, it does suggest that nurses need to be prepared to work with new technologies and digital solutions to provide high-quality care to patients. | Limited to English language papers published between 2011-2021, no quality assessment of included studies, potential publication bias. |
| 23 | Utilization of Robotics for Healthcare: A Scoping Review | Javaid; et al  (2022) | Journal of Industrial Integration and Management | Scoping Review /The authors conducted a comprehensive search of various databases to identify relevant studies on the utilization of robotics in healthcare. They then screened the studies based on inclusion and exclusion criteria and extracted data from the included studies to identify the various applications of robotics in healthcare. | Not Mention | Robatics and AI | -advancements in healthcare services  -The article discusses the use of various types of robotics technology in healthcare, including surgical robots, rehabilitation robots, telepresence robots, and autonomous mobile robots. | The article highlights the potential benefits of incorporating robotics into healthcare services, including improved patient outcomes, increased efficiency, and reduced workload for healthcare professionals. The authors also discuss the need for nurses to be trained in the use of robotics technology and to be involved in the development and implementation of robotics programs in healthcare settings. | The authors note that the scoping review methodology used in this study has some limitations, including the potential for publication bias and the lack of a formal quality assessment of the included studies. Additionally, the authors acknowledge that the use of robotics in healthcare is still in its early stages and that further research is needed to fully understand its potential benefits and limitations. |
| 24 | Nurse leaders' and digital service developers' perceptions of the future role of artificial intelligence in specialized medical care: An interview study | Laukka;et al  (2022) | J Nurs Manag | Descriptive qualitative methodology /This is a qualitative study that used semi-structured interviews to gather data from nurse leaders and digital service developers. | nurse leaders (n = 20) and digital service developers (n = 10) | AI in general | -improve clinicians’ workflows  - The study aimed to describe the potential impact of AI on work, care, services, and organizations in the healthcare industry. | The study suggests that AI will have a significant role in specialized medical care, but it will likely reinforce, rather than replace, clinicians or traditional care. Nurse leaders should be familiar with the potential of AI, but also aware of risks. Such leaders may provide better support for the development of AI-based health services that improve clinicians' workflows. | The study was conducted at a single university hospital in Finland, which may limit the generalizability of the findings. Additionally, the study did not include the perspectives of patients or other healthcare professionals, which could provide valuable insights into the use of AI in healthcare. |
| 25 | The Nursing Profession Needs to Adapt to the Digital | Millie; et al  (2022) | Perspective | The article discusses the benefits and challenges of digital technologies in nursing and provides recommendations for the profession to advance further in light of the findings | N/A | AI in general | N/A | The article suggests that AI technology could provide significant advantages in data analytics and sophisticated clinical decision support, which could support and extend nurses' cognitive, decision-making, and labor functions. However, the article also notes that there are concerns about the potential impact of AI on nursing, such as the possibility of perpetuating systemic disparities inherent in society. Overall, the article emphasizes the need for nursing to adapt to digital technologies, including AI, to maximize benefits to patient care, colleagues, and the profession. | The article does not provide specific data or research findings to support its recommendations. The recommendations provided may need to be qualified in light of regional context and professional background. |
| 26 | Explainable artificial intelligence (XAI): closing the gap between image analysis and navigation in complex invasive diagnostic procedures | Sullivan; et,al  (2022) | World Journal of Urology | Literature review /the literature search was performed on several databases, including PubMed/MEDLINE, Scopus, dblp, SSRN, Academia.edu, ResearchGate, and eur-lex.europa.eu. The review focused on articles that were published between the years of 2007 and 2021. All available publications were analyzed and summarized after an interdisciplinary collaborative review process. | NOT Mention | the article's title suggests that the focus is on explainable artificial intelligence (XAI) and its application in complex invasive diagnostic procedures. The introduction section briefly mentions that XAI is a subset of AI that aims to make the decision-making process of AI systems more transparent and understandable to humans. The article also discusses the importance of human control over AI systems in the context of medical decision-making. | improve clinicians’ practice | it does mention the importance of involving medical professionals, including nurses, in the development and implementation of AI systems in healthcare. The article emphasizes the need for human control over AI systems and the importance of ensuring that medical professionals remain responsible for decisions made based on AI recommendations. Additionally, the article discusses the potential benefits of AI-based medical devices in improving patient care, which could be relevant to nursing practice. | The article also emphasizes the importance of involving medical professionals in the development and implementation of AI systems in healthcare, as well as the need for regulatory oversight to ensure patient safety. Overall, the article suggests that XAI has the potential to revolutionize the field of cystoscopy and improve patient outcomes, but that care/A ful consideration must be given to the ethical and regulatory implications of these new technologies. |
| 27 | Clinician Adoption of an Artificial Intelligence Algorithm to Detect Left Ventricular Systolic Dysfunction in Primary Care | Rushlow; et al  (2022) | Mayo Clinic Proceedings | Retrospective Analysis of an RCT | 48 practice sites of a US Midwest health system | AI-Enabled Electrocardiogram Algorithm | improve clinicians’ practice | Artificial intelligence has the potential to transform nursing practice by enabling the diagnosis of occult and early disease, improving diagnostic accuracy, patient triage, and clerical burden reduction. However, to impact human health, AI tools must be adopted by clinicians, including nurses. AI-enabled tools may require even higher technical proficiency, and computer literacy is an important predictor in the successful adoption of clinical decision support tools. Nurses can play a critical role in the adoption and implementation of AI tools in healthcare, as they are often the front-line clinicians who interact with patients and use technology to deliver care. | The study was conducted in a single healthcare system, and the retrospective design may have introduced selection bias. |
| 28 | Beyond technology: Can artificial intelligence support clinical decisions in the prediction of sepsis? | Scherer; et al  (2022) | Rev Bras Enferm | An observational retrospective cohort study/ Analysis of critical alarms predictors of clinical deterioration/sepsis for clinical decision making in patients admitted to a reference hospital complex | 122,703 alarms | Machine Learning (ML) models | Speed up assertive clinical decisions by nurses, optimizing time and specialized human resources | AI can support assertive clinical decisions by nurses, optimize time and specialized human resources, and encourage evidence-based and personalized care. Nurses should be included in the development of both bedside protocols and AI models. | Adaptation of protocols based on the target patients' profiles and involvement of the multi-professional team, especially the nurses, are prerequisites for the successful use of AI in clinical decision making. The ethical and moral issues related to patient outcomes will always be the responsibility of the teams, who know and are involved with people, beyond the technology. |
| 29 | Can nurses in clinical practice ascribe responsibility to intelligent robots? | Tabudlo;et al  (2022) | Nurs Ethics | The authors present two worldviews, anthropocentrism and biocentrism, to respond to the question of whether nurses can ascribe responsibility to intelligent robots and AI when they commit errors. The article is written in a conversational and explanatory tone to illuminate a clear discussion of the involved concepts. | Not mention | AI in general | improve clinical practice | The article explores the ethical and moral implications of using intelligent robots and artificial intelligence in nursing and healthcare. The authors argue that nurses should engage in discussions and propose ethico-moral, efficient, and safe measures appropriate for the future. The article is the first in nursing to attempt to respond to the question of whether nurses can ascribe responsibility to intelligent robots and AI in clinical practice. | The article is a philosophical/theoretical paper and does not provide empirical evidence or data. |
| 30 | Artificial intelligence in health‐care: implications for the job design of healthcare professionals | Tursunbayeva and Renkema  (2022) | Asia Pacific Journal of Human Resources | The study used a systematic literature review to identify relevant articles on the impact of AI on healthcare job design. The authors analyzed the findings of the selected articles and synthesized them to identify the implications of AI for healthcare professionals' job design. | 80 publications | it does not discuss specific types of artificial intelligence. Instead, the article provides a framework to analyze AI applications in healthcare and their impact on healthcare professionals' job design. | improve clinical practice | The study revealed that AI has the potential to impact various aspects of healthcare professionals' job design, including diagnosis and treatment, patient engagement and empowerment, and administrative activities. The study provides insights into the potential implications of AI adoption for healthcare professionals' jobs, so they can prepare themselves for the future. Healthcare managers can start preparing their workforce to collaborate with AI systems and adjust the job design of healthcare professionals. | - |
| 31 | The Essence and Role of Nurses in the Future of Biomedical and Health Informatics | Weber;et al  (2022) | Stud Health Technol Inform | The authors conducted a literature review of relevant studies and reports to identify the current state of nursing and healthcare informatics. | Not mention | The article discusses the potential use of artificial intelligence (AI) in nursing and healthcare informatics, including the use of AI for decision-making and precision health. | improve clinical practice | The article highlights the need for nurses to develop adaptive and scientific skills in technology to improve healthcare delivery. It also emphasizes the importance of integrating new information technologies into practice to support practice improvements and improve quality of care. | - |
| 32 | Toward a Stronger Post-Pandemic Nursing Workforce | Buerhaus; et al  (2023) | New England Journal of Medicine | The authors reviewed the literature and discussed the challenges faced by the nursing workforce during the COVID-19 pandemic and the potential solutions to strengthen the nursing workforce post-pandemic. | Not mention | the authors mention the potential use of artificial intelligence (AI) to manage prioritization of care and other cognitive demands. | improve clinicians’ workflows | The use of AI technology in nursing can help to improve patient outcomes, reduce the burden on nurses, and enhance the efficiency of healthcare delivery. For example, AI can be used to analyze patient data and identify patterns that can help nurses make more informed decisions about patient care. AI can also be used to automate routine tasks, freeing up nurses to focus on more complex aspects of patient care. | - |
| 33 | Advancing Dermatological Care: A Comprehensive Narrative Review of Tele-Dermatology and mHealth for Bridging Gaps and Expanding Opportunities beyond the COVID-19 Pandemic | Giansanti  (2023) | Healthcare (Switzerland) | narrative review framework /The review considered papers written in English, along with two in German (available in Pubmed). Reviews in different languages which were not available in these databases were not considered. The PubMed and Scopus databases were consulted, and only peer-reviewed papers were considered in the review. Databases at the local/national level were not consulted. | 40 Articles | The review highlights the use of Artificial Intelligence (AI) and smartphone apps in tele-dermatology and mHealth for dermatological care. | improve clinical practice and managment | The review emphasizes the role of tele-dermatology and mHealth in providing remote specialist visits for citizens, in order to minimize face-to-face interactions. The use of AI and smartphone apps in dermatological care can provide important contributions, as they are used directly in the hands of the citizen, who has become an actor–operator–technologist at once with these developments. | The review has limitations as it only considered papers written in English and two in German (available in Pubmed). Reviews in different languages which were not available in these databases were not considered. Databases at the local/national level were not consulted. |
| 34 | Big Data in Oncology Nursing Research: State of the Science | Harris; et al  (2023) | Seminars in Oncology Nursing | review /Machine Learning Model using a "Bag of Words" Representation | 15 Articles | The article mentions the use of a machine learning model that used a "bag of words" representation to predict the needs expressed in each message. This is a type of natural language processing (NLP) technique that involves breaking down a text into individual words and analyzing them to identify patterns and relationships. In this case, the machine learning model was trained to identify different types of needs expressed in messages that referenced physical, psychological, social, and information needs. | Improve Nursing Practice | Nurses should be taught the definition, characteristics, applications, and limitations of big data. Nurse scientists who intend to collect and analyze big data should pursue training in the computational methods described in Papachristou et al's commentary on big data analytics. Nurses in all roles should be skilled at interdisciplinary collaboration. | - |
| 35 | Development of a deep learning-based tool to assist wound classification | Huang; et al  (2023) | J Plast Reconstr Aesthet Surg | Deep learning-based wound classification tool | 2149 wound images collected from 1429 patients randomly selected from the database of the corresponding author’s affiliated hospital | Convolutional neural network (CNN) | Improve Practice | Timely development of remote and intelligent diagnosis and prognosis systems for wound care, providing information about patients’ wounds to general practitioners, nurses, and even the patients themselves before seeing a wound specialist such that timely and efficient referrals can be achieved. | Generalizability of the proposed model, the model uses only the wound images to classify the wound tasks which might omit some other non-visual information. |
| 36 | Automated Healthcare System Using AI Based Chatbot | Mendon;et al  (2023) | Lecture Notes in Networks and Systems | The study utilizes a three-stage process, including data collection, pre-processing of text using natural language processing, and feeding the data to a machine learning model for training and testing. | Not mention | The chatbot utilizes Artificial Intelligence and Natural Language Processing to analyze patients' illnesses and provide essential insights regarding their infection. | Improve Practice | The chatbot can help reduce medical services costs, improve time management, technical resources, healthcare infrastructure, support staff, and healthcare personnel. Nurses can benefit from the chatbot by providing more efficient and accurate patient care. | The study's limitations include the need for further testing and validation of the chatbot's accuracy and effectiveness in real-world healthcare settings. Additionally, the chatbot's reliance on data collection and pre-processing may limit its ability to provide personalized care to patients. |
| 37 | Artificial Intelligence, Digital Health Research, and the Clinical Nurse Specialist | Siedlecki  (2023) | Clin Nurse Spec | The article explores how digital technology can be used to improve recruitment and retention of subjects in clinical trials and how it can be used to enhance data capture, providing a better and more complete picture of the results of interventions. | Not mention | AI combined with devices to enhance care and provide personalized care | Improve Practice | The clinical nurse specialist is encouraged to become versed in new interventions and new ways of collecting data to offer guidance and safeguard patients. The article highlights the importance of the clinical nurse specialist's clinical expertise and knowledge of nursing workflow in the development and refinement of digital interventions, digital content, and the use of sensor technology. | The article acknowledges that advances in digital health through artificial intelligence are complex and evolving at a very rapid pace, and that knowledge of artificial intelligence is essential to prepare the clinical nurse specialist to advocate for the rationale and ethical use of digital technology in clinical practice and research. |
| 38 | The future of care and healthcare provision to community-dwelling disabled elderly people in an ageing society | Susło; et al  (2023) | Family Medicine and Primary Care Review | A literature review/The authors conducted a literature review to identify the characteristics of modern technology-based tools for providing care and healthcare to community-dwelling people aged 60 years and older. | Not mention | it does mention the potential benefits of modern technology-based tools for providing care and healthcare to elderly people. These tools may include remote monitoring solutions that combine the potential of Internet of Medical Things (IoMT) sensors with non-medical devices like smartphones and wearables, as well as software for human activity recognition (HAr) and activities of daily life (ADL) and fall detection systems (FDS) that use artificial intelligence (AI) and deep learning. | Improve care | Some examples of AI in nursing include using machine learning algorithms to predict patient outcomes, using natural language processing to analyze patient data, and using robotics to assist with patient care. However, there are also concerns about the ethical implications of using AI in healthcare, such as issues related to privacy, bias, and accountability. | - |
| 39 | Digital Technologies for Public Health Services after the COVID-19 Pandemic: A Risk Management Analysis | Văduva; et al  (2023) | Sustainability (Switzerland) | Questionnaire-based survey conducted among nurses in various fields of medicine, such as cardiology, urology, internal medicine, and day hospitalization, working at “Prof. Dr. Theodor Burghele” Clinical Hospital in Bucharest. | questionnaire based  survey | The article mentions machine learning as one of the most important forms of AI that uses computational algorithms to evolve based on the lessons learned from previous experiences. Additionally, the article references other scientific articles that discuss the implications of AI and machine learning in healthcare. | Improve care | Digital tools introduced in the medical field show great importance both for medical specialists and patients, and they will increase in popularity as research and development continue. Most of the nurses are able to identify and classify the medical risks that may occur and efficiently deal with them. | The study has been conducted on a sample of 50 nurses, which may not be sufficient enough for a clear picture of the capacity of nurses to classify risks and their awareness of digital technologies implemented in hospitals for the purpose of risk management. A study with a larger sample has to be conducted in order to better understand the perception of nurses regarding digitalization in the public health sector and their ability to classify emerging risks in their field of activity. |
| 40 | Integrating Artificial Intelligence and Wearable IoT System in Long-Term Care Environments | Wang and Hsu  (2023) | Sensors (Basel) | The study develops an intelligent wearable physiological signal measurement integrated system that integrates multiple functional physiological signal measurement technologies into a device, including detection of heart rate, body temperature, blood oxygen, and blood pressure. It also combines wireless transmission technology to transmit physiological signals and location information back to the monitoring center in real time. | Not mention | The study utilizes AI algorithms and real-time data monitoring to provide personalized and timely interventions, ultimately leading to better health outcomes and a higher quality of care in long-term care settings. | Improve long term care management | The integration of AI and wearable IoT systems in long-term care environments offers significant opportunities for enhancing care delivery, promoting proactive health management, and improving the overall well-being of individuals. By leveraging the power of AI algorithms and real-time data monitoring, healthcare professionals can provide personalized and timely interventions, ultimately leading to better health outcomes and a higher quality of care in long-term care settings. | The study acknowledges that there may be technical challenges in ensuring the accuracy and reliability of wearable IoT devices. The calibration and validation of these devices are essential to ensure accurate data collection. Ongoing maintenance and monitoring of these devices are also necessary to detect any malfunctions or deviations that may affect the data quality. It is crucial to address privacy concerns, ensure data accuracy, and address technical challenges to fully realize the potential of this integration in long-term care environments. |
